# Network-Based Selection of Candidate Markers and Assays to Assess the Impact of Oral Immune Interventions on Gut Functions

**DOI:** 10.3389/fimmu.2019.02672

**Published:** 2019-11-13

**Authors:** Marjolein Meijerink, Tim J. van den Broek, Remon Dulos, Jossie Garthoff, Léon Knippels, Karen Knipping, Lucien Harthoorn, Geert Houben, Lars Verschuren, Jolanda van Bilsen

**Affiliations:** ^1^TNO, Zeist, Netherlands; ^2^Danone Food Safety Center, Utrecht, Netherlands; ^3^Danone Nutricia Research, Utrecht, Netherlands; ^4^Utrecht Institute of Pharmaceutical Sciences, Utrecht University, Utrecht, Netherlands

**Keywords:** gut assays, biomarkers, safety assessment, efficacy assessment, systems biology, immune intervention, network databases

## Abstract

To assess the safety and efficacy of oral immune interventions, it is important and required by regulation to assess the impact of those interventions not only on the immune system, but also on other organs such as the gut as the porte d'entrée. Despite clear indications that the immune system interacts with several physiological functions of the gut, it is still unknown which pathways and molecules are crucial to assessing the impact of nutritional immune interventions on gut functioning. Here we used a network-based systems biology approach to clarify the molecular relationships between immune system and gut functioning and to identify crucial biomarkers to assess effects on gut functions upon nutritional immune interventions. First, the different gut functionalities were categorized based on literature and EFSA guidance documents. Moreover, an overview of the current assays and methods to measure gut function was generated. Secondly, gut-function related biological processes and adverse events were selected and subsequently linked to the physiological functions of the GI tract. Thirdly, database terms and annotations from the Gene ontology database and the Comparative Toxicogenomics Database (CTD) related to the previously selected gut-function related processes were selected. Next, database terms and annotations were used to identify the pathways and genes involved in those gut functionalities. In parallel, information from CTD was used to identify immune disease related genes. The resulting lists of both gut and immune function genes showed an overlap of 753 genes out of 1,296 gut-function related genes indicating the close gut-immune relationship. Using bioinformatics enrichment tools DAVID and Panther, the identified gut-immune markers were predicted to be involved in motility, barrier function, the digestion and absorption of vitamins and fat, regulation of the digestive system and gastric acid, and protection from injurious or allergenic material. Concluding, here we provide a promising systems biology approach to identify genes that help to clarify the relationships between immune system and gut functioning, with the aim to identify candidate biomarkers to monitor nutritional immune intervention assays for safety and efficacy in the general population. This knowledge helps to optimize future study designs to predict effects of nutritional immune intervention on gut functionalities.

## Introduction

A well balanced immune system is key for overall health and well-being, therefore the concept of boosting immunity is gaining in popularity as today's complex world presents many potential health challenges. These health challenges range from environmental pollution, infections, the use of medication, the harmful effects of lifestyle stress and the effects of intense physical workouts on the body's natural ability to stay healthy. Risk reduction measures or immune health interventions may be effective for reducing the loss of health, loss of quality of life as well as the costs to society and health care due to immune related diseases and disorders.

Besides beneficial effects of oral immunotherapy and –prophylaxis on the functioning of the immune system itself (1–10), (re/un) balancing the immune system may also generate dis-immune toxicities. These so-called “immune-related adverse events” can come forward in the immune system itself [recently addressed in (11)], resulting in, for instance, increased incidences or severity of inflammatory diseases, but may also affect other immune system-interacting organ systems. The gut being the porte d'entrée of oral immunotherapy has been selected as one of the key priority organs to include in this study. However, despite established association studies that immunotherapy can influence gut functioning (12–16), it is still largely unknown which immune and gut function-related pathways and biomarkers are crucial to monitor in relation to gut functioning upon nutritional immune interventions.

Previously, immune cells and immune cell mediators have been described as affecting gut functions in several studies. Yu et al. showed that the mucosa of the gastrointestinal tract contains large numbers of immunocompetent cells, including mast cells, lymphocytes and granulocytes (17). These mast cells play an important role in normal physiology functions (regulation of vasodilation, vascular homeostasis, innate and adaptive immune responses, angiogenesis, and venom detoxification) and pathophysiology (including allergy, asthma, anaphylaxis, gastrointestinal disorders, many types of malignancies, and cardiovascular diseases) (18). For instance, mast cells are known to be involved in gastrointestinal motility, abdominal pain, discomfort, and gut barrier function. Mast cells are present in all compartments of the gastrointestinal (GI) tract. Upon activation, they release an array of inflammatory mediators including histamine, 5-hydroxytryptamine (5-HT), neutral proteases (tryptases, chymases, and carboxypeptidase A), prostaglandins, leukotrienes, platelet activating factor (PAF), and several cytokines including tumor necrosis factor-α (TNF-α), interleukin (IL)−3,−4,−5,−6 and granulocyte macrophage colony stimulating factor (GM-CSF) (17). When the gut is sensitized e.g., in case of irritable bowel syndrome, the infiltration of mast cells and the release of mediators are proven to be associated with disturbed motility (19). The motility is disturbed by, for example, an increase in colonic and intestinal myoelectric spike activity (20), the contraction of circular and longitudinal smooth muscle (21), and intense duodenal clusters of contraction (22). Additionally, mast cells have shown to play a role in chronic pain, mainly at the visceral level (23). There is also a positive relationship between the intestinal permeability and the number of mast cells with diarrhea predominant irritable bowel syndrome (24). The mast cell derived tryptase has been identified as a key factor that disrupts the intestinal barrier (25). Additionally mast cell mediators, such as interferon-γ (IFN-γ), TNF-α, IL-1β, IL-4, IL-13, and prostaglandin E2 (PGE2), have been shown to have destructive effects on both trans- and paracellular permeability (26).

Aside from mast cells, other immune cells and immune cell mediators can also affect gut functions, such as the intestinal barrier function. Multiple factors regulate the intestinal barrier, including exogenous factors, epithelial apoptosis, cytokines, and immune cells. Immune-induced intestinal barrier dysfunction is thought to be critical in the predisposition to and exacerbation of numerous autoimmune and inflammatory conditions, including inflammatory bowel disease (IBD), food allergy, celiac disease and diabetes (27, 28). Several mucosal immune cells have been implicated in the breakdown of intestinal barrier function such as gamma/delta-positive intestinal intraepithelial (iIELγδ+) T cells (29) and eosinophils (28). Their mediators, such as IFN-γ, TNF-α and some eosinophil granular proteins (e.g., major basic protein, eosinophil peroxidase, and eosinophilic cationic protein), promote the reorganization of several tight junction (TJ) proteins (e.g., zonula occludens-1, junctional adhesion molecule A, occludin, claudins-1 and−4) (30) or their expression (31), all resulting in a decreased epithelial barrier function. In contrast, IL-10 has been shown to positively regulate intestinal barrier function (32).

Food transit is another important feature of gut function. Food transit is influenced by the gastrointestinal motility which can be altered by inflammation and immune activation. Though the innate immune response does not seem to have a major role in muscle function, animal studies have shown that a Th1 immune response is associated with hypocontractility (33) and a Th2 immune response with hypercontractility (34) of inflamed intestinal smooth muscle (35). It has been reported that IL-1β plays an important role in decreased GI smooth muscle contractility in Th1 cytokines-dominant colitis, by downregulating C-kinase-activated protein phosphatase-1 (PP1) inhibitor, 17 kDa (CPI-17) expression (36–38). The inhibitory effect of IL-1β on the GI smooth muscle contraction can also be mediated by the upregulation of regulator of G protein signaling 4 (RGS4) expression by inhibiting NF-κB activation (38, 39). Th2 cytokines may have opposing mechanisms to downregulate RGS4 expression.

These examples illustrate that the risks and benefits of restoring or changing the immune balance by novel treatment strategies of immune-related disorders are not limited to immune resistance or the inflammatory status as such, but that these immune interventions can also impact gut physiology.

The aim of this study is to illustrate how systems biology can help in clarifying the relationships between the immune system and gut function, as well as the identification of crucial biomarkers to monitor effects on gut functions upon nutritional immune interventions in the general population. In this paper we will focus on effects of immunonutrition on five major physiological functions of the gut: transport/transit of ingested material, extracellular digestion of ingested material, intracellular digestion and metabolism, uptake of essential nutrients, and protection from injurious or allergenic material.

## Materials and Methods

### Literature Study on the Immune System—Gut Function Relationships

An inventory of the available literature regarding the relationship between the immune system and gut functioning was performed. The following biomedical databases were searched: CAB ABSTRACTS, Embase^®^, MEDLINE^®^, Current Contents^®^ Search, and BIOSIS Previews^®^. The databases were searched between 15th of February 2017 and 15th of March 2018.

The following search string was used: ti,ab ((immune^*^ OR immuno^*^ OR immuni^*^) AND ((intestin^*^ n/3 function) OR (gut P/0 barrier) OR (intestin^*^ P/0 barrier) OR (gut P/0 integrity) OR (intestin^*^ P/0 integrity) OR ((gut OR intestin^*^) AND (nutrient^*^ P/0 absorption)) OR (intestin^*^ p/0 brush p/0 border) OR (gut p/0 brush p/0 border) OR ((gut OR intestin^*^) AND (peristalsis OR motility Or peristaltic)) OR ((gut OR intestin^*^) AND (water p/0 absorption)) OR ((gut OR intestin^*^) AND (transit p/0 time))) NOT (HIV OR “immunodeficiency virus” OR AIDS OR “acquired immunodeficiency syndrome” OR “cell line” OR dog OR dogs OR canine OR cat OR cats OR feline OR horse OR horses OR cattle OR animal OR animals OR veterinary OR broiler OR broilers OR chick OR chicks OR chicken. The following criteria were applied to obtain the most relevant hits and reduce number of hits: 2007–2018; English; Excluding: Conference Abstract, Editorial Material, Editorial, Book Chapter, Short Survey, Conference Review, Letter, Chapter, Meeting Poster, Note, Erratum, Patent, Book, Corrected and Republished Article, Correction, Correspondence, Meeting Summary, Published Erratum, Thesis, News.

During the selection process of relevant manuscripts describing the interaction between the immune system and gut function, information from manuscripts was collected that describe several key functions and clinical endpoints of the gut. In addition, several guidance documents were studied to identify those key functions and clinical endpoints of the gut that are usually indicated by the regulatory authorities (40–42).

### Selection of Candidate Biomarkers Linking the Immune System to Gut Functionality

First, the different gut functionalities were categorized based on literature and EFSA guidance documents, and an overview of the current assays and methods to measure gut function was generated. Secondly, related adverse events and biological processes were selected based on literature and expert knowledge and linked to the physiological functions of the GI tract. Thirdly, database terms and annotations from Gene Ontology [GO; (www.geneontology.org/) and CTD (http://ctdbase.org/); (43)] databases were selected that were related to the biological processes as identified previously. Next, key pathways involved in those gut functionalities and the involved genes were retrieved from the GO and CTD databases: The GO database links genes to processes (so called GO terms) related to gut function. The CTD database—enabled us to connect genes/proteins to diseases which are uniquely identified by MeSH identification numbers (so called MeSH IDs). Next, the gut-function related genes were compared with the previously identified set of immune health related genes (11). Neo4J is a graph-database with query-based calculations (Neo4j, Inc., San Mateo, CA, USA) and is used, together with Venn-diagrams (44) to visualize and calculate the unique and overlapping genes/proteins among the health endpoints/processes. Using bioinformatics enrichment tools DAVID 6.8 [https://david.ncifcrf.gov; bioinformatics resources using Kyoto Encyclopedia of Genes and Genomes (KEGG) pathway output, updated 2019] and Panther 14.1 [web-based software; www.pantherdb.org/pathway, updated 19th December 2018; (45)], the identified gut-immune markers were evaluated by predicting the effects of these gut-immune genes on gut functions. DAVID was used as tool to display the candidate biomarkers on pathway maps from KEGG to facilitate the biological interpretation in a network context. To this end, the gut-immune genes associated with a specific gut function were entered into DAVID after which the top 15 KEGG pathways related to the entered genes were retrieved.

**Figure 1:**
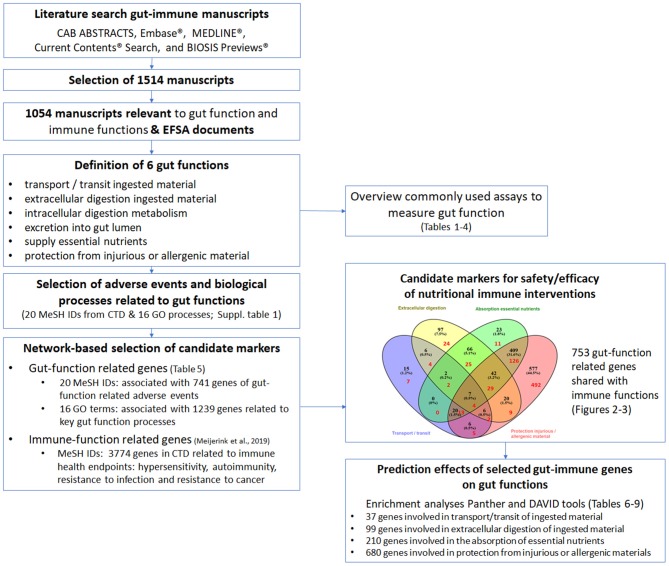
Schematic overview of the work flow to identify gut-function related genes possibly affected by immune interventions and to identify commonly used assays to measure gut function. The literature search resulted in a total of 1,514 manuscripts, of which 1,054 manuscripts were considered relevant to gut and immune functions. The information from the 1,054 manuscripts was collected resulting in the definition of six key gut functions. An overview was made of the commonly used assays to study gut function (depicted in Tables 1–4). The adverse events related to malfunctioning of the gut and key biological processes related to the gut functions were selected from the databases Comparative Toxicogenomics Database (CTD; 20 MeSH IDs) and GeneOntology (GO; 16 GO processes). Next the genes associated with the 16 GO processes and 20 MeSH IDs were retrieved from respectively GO (1,239 genes) and CTD (741 genes). Thereafter, CTD was used again to retrieve 3,774 immune disease related genes of the immune health endpoints hypersensitivity, autoimmunity, resistance to infection and resistance to cancer, as described previously (11). In total 753 genes were predicted to be involved in both gut functions and immune functions, indicating the strong relationship between the immune system and the gut functions. To study which biological processes in the different gut functions are predicted to be influenced by the 753 gut-immune related genes, a GO enrichment analysis (Panther web-based software; www.pantherdb.org/pathway) and an enrichment analysis using DAVID (bioinformatics resources using Kyoto Encyclopedia of Genes and Genomes (KEGG) pathway output) was performed, resulting in a top 15 selection of processes and pathways per gut function (depicted in Tables 6–9).

Panther was used as tool to identify GO processes shared between the gut and immune system. Hereto, the gut-immune genes associated with a specific gut function were entered into Panther after which the top 15 GO processes related to the entered genes were retrieved. Figure 1 depicts the workflow and steps for the identification of potential candidate markers.

### Proof of Principle/*in silico* Test Case

After selecting a large set of genes involved in the four gut functionalities, the next step was to check whether this set of genes could be validated by predicting whether an oral immune intervention can result in a disturbance of any of the gut functionalities. To this end, vitamin D was selected because it is known for its effects on (i) the immune response and (ii) adverse/beneficial effects on gut function are described, and (iii) top interacting genes are described in the CTD. The CTD contains curated data on the top interacting genes affected by a chemical/food substance. Using these curated data, the top interacting genes were compared with our previous set of gut-function related genes, to predict the effects on those gut-functionalities after which it was checked whether the predicted gut-effects could be confirmed in previously described adverse/beneficial effects on gut functionalities.

## Results

### Literature Study on Immune System—Gut Function Relationships

The literature study resulted in a total of 1514 articles which were reviewed. From these, 30.4% (460) were rejected and 69.6% (1, 54) were considered relevant. The reasons for rejections were: article not in English, species with low physiological similarity toward human GUT/immune system (e.g., fish, horses) or not the right focus. Of the remaining articles, 54.5% (575) were review articles and 44.5% (479) were original research articles. During the selection process of relevant articles about the interaction between immune system and gut function, information describing key functions and clinical endpoints of the gut was collected. In addition, several guidance documents were studied to identify those key functions and clinical endpoints of the gut that are usually specified by the regulatory authorities.

### Key Gut Functions and Gut Assays to Study the Effects of Oral Immunotherapy

All collected clinical endpoints and coinciding analyzed parameters from literature and guidance documents were structured (see Tables 1–4). Based on this, we propose that the possible effects of immune interventions should be measured on the following four major physiological functions of the gut using the corresponding currently used assays:

1. *Transport and transit of ingested material (Table 1):*

The function of the healthy small intestine is to transport food, to mix it with bile and with pancreatic and intestinal secretions to facilitate digestion and absorption over the intestine mucosal surface. When there is a disturbance in gut health, common symptoms are abdominal pain or discomfort, diarrhea, constipation, fullness, and bloating. The underlying cause of constipation consists of poor intake of fluid or fiber, slow colonic transit, and outlet dysfunction in the anorectal area. Diarrhea is an increase in the volume of stool by fluid with or without frequency of defecation, and is categorized into osmotic, secretory, exudative, and altered intestinal motility. In addition, vomiting, reflux, and regurgitation are also adverse events related to this gut physiological function.

**Table 1:** Methods described in literature to measure transport and transit ingested material.

**Gut function**	**Process**	**Method**	**Read out**	**Applicable sites**	**Biological samples**	**Example references**
Transport/transit	Transit	Fluorescent microscopy of mounted tissue *ex vivo*	Trypan blue staining in tissue	Small intestine	Tissue samples	(46)
		Fluorescent microscopy of mounted tissue *ex vivo*	Carmine red solution staining	Small intestine	Feces	(47)
		ELISA	Fluorescent-labeled dextran/rhodamine B-conjugated dextran	Whole intestine	Plasma	(48)
		Whole-mount preparation of the myenteric ganglia	NOS-IR neurons staining in tissue	Small neural intestine	Tissue samples	(49)
		Fluorescent microscopy of mounted tissue *ex vivo*	GpBAR1-IR expression in tissue	Whole intestine	Tissue samples	(50)
		Bead expulsion test	Transit time glass ball	Colon	na, *in vivo* test	(51)
		X-ray contrast examination	Labeled barium (meal) transfer in gastrointestinal tract	Gastrointestinal tract	na, *in vivo* test	(52)
	Peristalsis/motility	Smooth muscle cell contractility by exposing the muscle strip to e.g., increasing concentrations of the muscarinic agonist bethanechol	Electrical field	Whole intestine	Tissue samples	(53)
		Immunohistochemistry	Neuronal nitric oxide synthase	Whole intestine	Tissue samples	(54)
		Immunohistochemistry	tyrosine kinase receptor (c-kit) and serotin	Small intestine	Tissue samples	(55, 56)
		Analysis by reversed phase high performance liquid chromatography with fluorometric detection	5-HT and 5-HIAA	Whole intestine	Platelet poor/depleted plasma or urine/	(57)
		ELISA	GLP-1, PYY and SS (three GI motility-inhibiting peptides)	Whole intestine	Plasma	(58)
		ELISA	Vasoactive intestinal peptide (VIP)	Whole intestine	Plasma	(59)
		Quantification by real-time PCR	GLP-1R expression in tissue	Colon	Tissue samples	(60)
		Contractility of bowel	Frequency of bowel movements (*ex vivo*)	Jejunum/colon		(61)
		ELISA	Motilin	Whole intestine	Plasma	(62)
		ELISA	Neuropeptide Y	Whole intestine	Plasma	(58)
	Defecation	Consistency of stools	Bristol Stool Form Scale (visual guide for stools)	na	Feces	(63, 64)
		Stool water content	Water content	na	Feces	(63, 64)
		Defecation pattern	Frequency of defecation	na	Feces	(63, 64)

Frequently used methods to measure transit include dyes [e.g., trypan blue (46), carmine red solution (47), fluorescent labeled dextran or rhodamine B-conjugated dextran (48)] which after oral gavage the amounts are measured in tissue samples. In addition bead expulsion test (in animals) (51), X-ray contrast examination with barium meal (52) and fluorescent microscopy of whole mount preparation of the myenteric ganglia to study BIS-IR neurons (49) or GpBAR1-IR expression (50) are used to study (in)directly transit. The peristalsis/motility is often measured by smooth muscle cell contractility by exposing the muscle strip to e.g., increasing concentrations of the muscarinic agonist bethanechol (53), or indirectly by immunohistochemistry by measurement of neuronal nitric oxide synthase (54) and tyrosine kinase receptor (c-kit) and serotine (55, 56), ELISA by measurement of 5-HT and 5-Hydroxyindoleacetic acid (5-HIAA; in platelet poor/depleted plasma or urine) (57) or glucagon like peptide-1 (GLP-1), peptide YY (PYY) and somatostatin (58), vasoactive intestinal peptide (59), motilin (62), and neuropeptide Y (58) in plasma and quantification of GLP-1-Receptor (60) by real time PCR in the colon. Also the frequency of bowel movements of the jejunum and colon are often checked in animals (61).

The defecation pattern can be monitored using a consistency score of stools (e.g., Bristol Stool Form Scale), by measurement of the stool water content, frequency of defecation and production fecal pellets (63, 64). The gastrointestinal symptom score can be used for the assessment of a broad spectrum of gut related symptoms including included nausea, vomiting, bloating, abdominal cramps, early satiety, acidic eructation/heartburn, loss of appetite, retrosternal discomfort, and epigastric pain/upper abdominal pain (72).

2. *Extracellular digestion of ingested material (Table 2):*

Extracellular digestion is the process by which food is converted into substances that can be absorbed and assimilated. This is accomplished in the digestive tract by the mechanical and enzymatic breakdown of food constituents into simpler chemical compounds. Imbalances in extracellular digestion can lead to a level of GI discomfort, nutritional imbalances and allergy and infection susceptibility.

**Table 2:** Methods described in literature to measure extracellular digestion.

**Gut function**	**Method**	**Read out**	**Applicable sites**	**Biological samples**	**Example references**
Extracellular/intracellular digestion	Indirect calorimetry	Oxygen uptake and carbon dioxide production	Whole intestine	Breath	(65)
	ELISA	Ghrelin	Whole intestine	Plasma	(66)
	Gas chromatography/mass spectrometry	SCFAs and branched chain fatty acids	Whole intestine		(67)

Methods to assess extracellular digestion include indirect calorimetry (65), ELISA by measurement of ghrelin (66) in plasma and gas chromatography or mass spectrometry to measure short-chain fatty acids (SCFAs) and branched fatty acids, which are produced by the gut microbes by fermentation (67).

3. *Absorption of essential nutrients (Table 3):*

Digested molecules of food, water and minerals from the diet are absorbed from the lumen of the upper and middle small intestine. The absorbed materials cross the mucosa into the blood or lymph and are carried off in the bloodstream to other parts of the body for storage (e.g., as energy source) or further chemical change. This process varies with different types of nutrients. Imbalances in absorption can lead to nutritional imbalances, disturbed defecation patterns and increased levels of GI discomfort.

**Table 3:** Methods described in literature to measure the supply of essential nutrients.

**Gut function**	**Method**	**Read out**	**Applicable sites**	**Biological samples**	**Example references**
Supply essential nutrients	Nutrient absorption	Blood galactose assay	Whole intestine	Blood	(68)
	Meal-stimulated, endogenous GLP-2 secretion	GLP-2 ELISA	na	na, *in vivo*	(64, 69)
	Metabolic chambers	Ratio of carbon dioxide and oxygen along with nitrogen	Whole intestine	Breath	(70)
	Carbohydrate malabsorption test	Hydrogen (product colonic bacterial fermentation)	Colon	Breath	(71)
	14C-triolein test: dose of a carbonyl-labeled triglyceride added to a fatty meal	^14^CO_2_	Whole intestine	Breath	(71)
	Plasma/blood levels	Vitamins (A, D, E, B12) Elements (Mg, Zn, Se, Cu, Mn) Iron Lipids	Whole intestine	Blood/plasma	(71)
	Symptom score absorption	Dyspeptic and malabsorption symptoms	na	na, *in vivo*	(72, 73)
	Fecal fat test	72-h stool for fecal fat test	Whole intestine	Feces	(71)

Methods to assess the supply of essential nutrients are for instance blood galactose assay for measurement of *in vivo* nutrient absorption (68), the measurement of meal-stimulated, endogenous glucagon-like peptide-2 (GLP-2) secretion (64, 69). In plasma/blood, levels of vitamins, minerals, iron and lipids can be determined (71), whereas in breath hydrogen (measure for carbohydrate malabsorption as a product of colonic bacterial fermentation) (71) and carbon dioxide (measure for triglyceride uptake) (71) are often used. In addition metabolic chambers (70) are used to measure the ratio of carbon dioxide and oxygen along with nitrogen. Urine is also frequently collected to assess for instance protein oxidation. Symptom score for dyspeptic and malabsorption symptoms are also often used (72, 73).

4. *Protection from injurious or allergenic material (Table 4):*

In the intestinal tract, a single layer of epithelial cells forms a physical barrier between the intestinal lumen, the lamina propria and the mucosal-associated lymphoid tissue. In addition, mucus secreted by goblet cells in the epithelium serves to spatially compartmentalize the bacteria to the lumen and prevent bacterial colonization of the epithelium. Increased mucosal permeability and loss of epithelial integrity are recognized to play a role in the pathophysiology of a variety of (gastrointestinal) disorders and possibly allergy, including the challenge by intestinal pathogens. Similarly, the defecation pattern and the level of GI discomfort is affected by an impaired protection.

**Table 4:** Methods described in literature to measure protection from injurious or allergenic material.

**Gut function**	**Process**	**Method**	**Read out**	**Applicable sites**	**Biological samples**	**Example references**
Protection	Intestinal permeabiltiy	Measurement of short circuit current in Ussing chambers	Ion transport, FD4 and HRP flux	Whole intestine	Biopsies	(74)
		Microsnapwell assay	Transepithelial electrical resistance	Whole intestine	Tissue samples	(75)
		Everted gut sac system	Mannitol	Whole intestine	Tissue samples	
		Dual sugar quantification using mass spectrometry	Oligosaccharides of different MW (e.g., lactulose/mannitol)	Small intestine	Urine	(76)
		Quantification using mass spectrometry	PEGs, 4,000/400 kDa	Whole intestine	Urine	(74, 76, 77)
		^51^Cr-EDTA radioisotope activity	^51^Cr-EDTA	Whole intestine	Urine	(78)
		LAL assay	Endotxin (LPS)	Whole intestine	Plasma	(79)
		Enzyme Immunoassay	Diamine oxidase (DAO) activity			(80)
		Modified spectromety	D-lactate	Whole intestine	Plasma	(80)
		Isolated intestinal loops	Non-digestible markers, labeled bacterial products or live bacteria	Small intestine	Tissue samples	(81)
	Epithelial integrity and intestinal inflammation	Mass spectrometry	Citrulline (an epithelial amino acid not incorporated into protein)	Whole intestine	Plasma	(82)
		ELISA	I-FABP	Jejunum	Plasma	(83–85)
		ELISA	I-BABP	Ileum	Plasma	(83–85)
		ELISA	L-FABP	Whole intestine	Plasma	(83–85)
		ELISA	Alpha-1-Antitrypsin (A1AT)	Whole intestine	Plasma	(86)
		ELISA	Zonulin, claudin 3 (potentially other junction proteins)	Whole intestine	Plasma	(87–89)
		Confocal fluorescence microscopy of TJ proteins and CLD-1	TJ proteins and CLD-1	Whole intestine	Biopsy or surgical tissue	(90, 91)
		ELISA	Antimicrobials			
		ELISA	Calprotectin	Whole intestine	Feces	(92, 93)
		ELISA	LCN-2	Whole intestine	Feces	(94)
		Quantification by real-time PCR	miRNAs upregulated in inflamed enterocytes	Whole intestine	Feces or plasma	(95)
	Mucus thickness and penetrability	Morphological studies using paraffin fixed tissue	Tissue appearance and morphology	Whole intestine	Biopsy or surgical tissue	(96)
		Fluorescent microscopy of mounted tissue *ex vivo*	Permeability of fluorescent beads through mucus	Whole intestine	Biopsy or surgical tissue	(96)
		Carnoy fixation and mucus detection using PAS/Alcian blue or antibodies	Secreted mucus	Whole intestine	Tissue samples	(96)
		Fluorometric assay kit	Fecal mucin content	Whole intestine	Feces	(96)
		Light microscopy	Goblet cells	Whole intestine	Biopsy or surgical tissue	(96)

Permeability of the small intestine is commonly evaluated by measurement of intestinal permeation and urinary excretion of orally administered water-soluble, non-metabolizable sugars that differ in size [e.g., lactulose, FITC-dextran or polyethylene glycols (PEGs) of 1,500–4,000 Da] and low-molecular-weight sugars such as mannitol and L-rhamnose, or low-molecular-weight PEG (400 Da) (74, 76, 77, 97). The ^51^Cr-EDTA test, which is performed by calculating the percentage of recovery from urine of an oral dose of ^51^Cr-EDTA, is also used as a permeability test (78). Another powerful model to assess intestinal permeability are creating isolated intestinal loops (81). Diamine oxidase (DAO) activity is measured in serum as it correlates inversely with intestinal permeability of the small intestine (80). Alpha-1-Antitrypsin (A1AT) in plasma and stool can also be used as a biomarker of intestinal permeability (86).

Potential biomarkers of epithelial integrity are fatty acid-binding proteins (FABPs) (83–85), TJ proteins (87–89), and citrulline (82) which can be measured in plasma and urine using ELISA. Detection of the inflammatory markers calprotectin (92, 93) and lipocalin 2 (94) in feces have also been used as a surrogate marker of epithelial integrity in many disease studies because excessive intestinal inflammation is known to increase epithelial permeability. Recently, non-coding microRNAs (miRNAs) such as miRNA-222, miRNA-30, miRNA-29b, miRNA-503, miRNA195, and miRNA-320a have been demonstrated to play a role in the regulation of epithelial regeneration, protection, and epithelial barrier function (95).

Ussing chamber (74) or microsnap well (75) are used to measure the short-circuit current which is an indicator of ion transport across the epithelium and to confirm the integrity and permeability using biopsies of patients and healthy subjects. Histological examination using biopsies or resected tissue from animals and humans is also a common experimental approach for studying aspects of barrier function by for instance immunofluorescent antibody detection of TJs (90) or adherens junctions (91) or to detect intestinal pathology including ulcerations of the mucosa and severe intestinal inflammation that will contribute to increased intestinal permeability. Carnoy fixation and paraffin embedding of intestinal tissue followed by immunofluorescent antibody staining of mucin Muc2 or periodic acid Schiff/Alcian blue staining can be used to assess mucus thickness in the colon of small rodents (96).

As shown, the symptoms of GI disorders reflect a broad spectrum of disturbance of GI physiology, including altered epithelial, muscle, intestinal, and enteric neural function which are at least in part, associated with immune activation. There are multiple assays available to address changes in gut functions but most emphasis is on transport and transit of ingested material and protection from injurious or allergenic material.

### Selection of Candidate Biomarkers Linking the Immune System to Gut Functionalities

Based on the identified gut functions (transport/transit ingested material, extracellular digestion ingested material, intracellular digestion and metabolism, absorption essential nutrients and protection from injurious or allergenic material), the next step was to select candidate molecules that are affected by immunotherapy that might influence gut functions. Therefore, molecular databases were used to connect genes/proteins involved in gut functions using Gene Ontology/Panther database and CTD database (Supplementary Table 1). In Table 5 the results are shown for each different gut health function. In total 741 unique molecules were found using the CTD MeSH terms and 1,239 unique molecules using the Gene Ontology database, of which 100 molecules were overlapping.

**Table 5:** Number of identified genes that are related to a gut function using the Comparative Toxicogenomics Database (CTD) and Gene Ontology (GO) database.

**Healthy gut functions**	**CTD MeSH term-related genes**	**GO term-related genes**
Transport/transit ingested material	43	31
Extracellular digestion ingested material	94	206
Intracellular digestion and metabolism	Not identified	664^*^
Excretion Into gut lumen	Not identified	Not identified
Supply essential nutrients	113	475
Protection from injurious or allergenic material	804	465

* *Excluding GO term “cellular metabolic process” (too broad)*.

**Figure 2:**
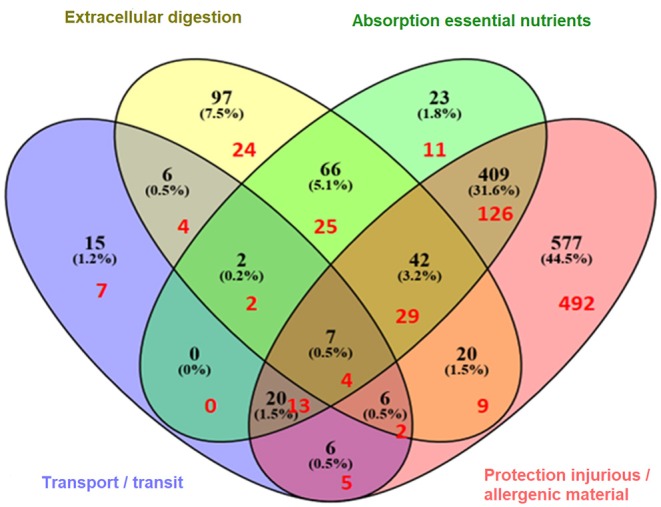
Venn diagram illustrating the (shared) sets of molecules involved in gut function-related processes: extracellular digestion ingested material, supply essential nutrients, transport/transit of ingested material, and protection from injurious or allergenic material (number of genes in black). In red are indicated the number of genes that are predicted to be shared with immune related diseases (hypersensitivity, autoimmunity, resistance to infection, and cancer) as previously described (11).

Next, the overlap of genes with multiple gut functions was evaluated and visualized in a Venn diagram (Figure 2). The names of the individual genes per gut function are depicted in the Supplementary Tables 2–5. Seven genes were overlapping between all gut functions: *GUCY2C, EPCAM, MYO5B, PYY, SLC10A2, TNF*, and *NEUROG3*.

**Figure 3:**
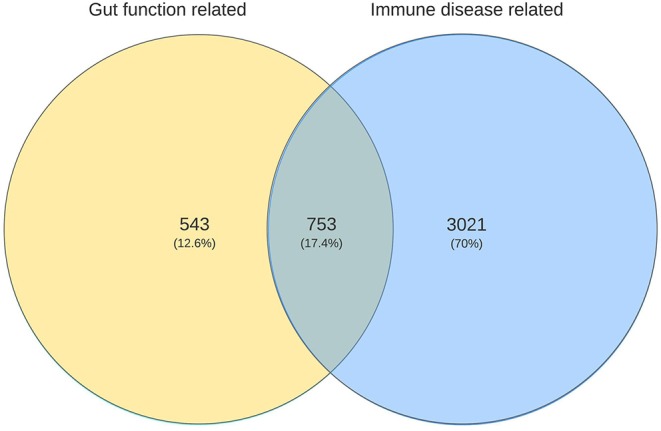
Venn diagram illustrating the numbers of unique and shared genes related to gut functions with immune disease related genes.

As referred to earlier, using the same approach as here, immune related genes were identified which are predicted to be involved in the four most common immune health endpoints:: hypersensitivity, infection, autoimmunity, and immune-mediated resistance to cancer (11). The overlap between the gut function related genes and immune disease related genes was determined revealing that in total 753 genes were shared (Figure 3), indicating the strong relationship between the immune system and gut function. These shared genes were evaluated and visualized in Figure 2 (indicated in red). The number of shared genes was as follows: transport/transit ingested material [37 out of 62 transport-related genes shared (60%)], extracellular digestion ingested material [99 out of 246 digestion-related genes shared (40%)], absorption essential nutrients [210 out of 569 absorption-related genes shared (37%)], and protection [680 out of 1100 protection-related genes shared (62%)]. Of the seven genes that were overlapping between all gut functions (*GUCY2C, EPCAM, MYO5B, PYY, SLC10A2, TNF*, and *NEUROG3*, as described previously) four genes were also immune disease related genes: *GUCY2, EPCAM, MYO5B*, and *TNF*.

### Prediction of Effects of Selected Gut-Immune Genes on Gut Functions

To study which biological GO processes and KEGG pathways in the different gut functions are predicted to be influenced by the immune system, a GO enrichment analysis was performed using Panther 14.1 and an KEGG pathway enrichment analysis using DAVID 6.8 were performed using the 753 gut-function related genes which are shared with the immune disease related genes.

#### Predicted Effects on Transport/Transit of Ingested Material

The 37 genes involved in transport/transit of ingested material which are shared with the immune system (Figure 2, red numbers in purple area) were used for the enrichment analysis. The top 15 biological GO processes and KEGG pathways involved in transport/immune are listed in Table 6. The biological processes in transport and transit are most likely to be affected in the regulation of the small intestinal transit, regulation of the hindgut contraction, regulation of the small intestine smooth muscle contraction, regulation of appetite and eating behavior, regulation of response to food, and regulation of gastric motility/gastric emptying.

**Table 6:** Top 15 GO processes and KEGG pathways related to the selected 37 gut-immune genes involved in transport and transit of ingested material.

**Top 15 GO processes**	**Top 15 KEGG pathways**
Positive regulation of small intestinal transit (GO:0120058)	Hematopoietic cell lineage
Regulation of small intestinal transit (GO:0120057)	Neuroactive ligand-receptor interaction
Regulation of hindgut contraction (GO:0043134)	Cytokine-cytokine receptor interaction
Positive regulation of small intestine smooth muscle contraction (GO:1904349)	Inflammatory bowel disease (IBD)
Regulation of small intestine smooth muscle contraction (GO:1904347)	Salmonella infection
Positive regulation of appetite (GO:0032100)	Influenza A
Positive regulation of response to food (GO:0032097)	Tuberculosis
Regulation of gastric motility (GO:1905333)	cAMP signaling pathway
Positive regulation of vitamin metabolic process (GO:0046136)	Graft-vs.-host disease
Positive regulation of CD4-positive, CD25-positive, alpha-beta regulatory T cell differentiation (GO:0032831)	Type I diabetes mellitus
Regulation of CD4-positive, CD25-positive, alpha-beta regulatory T cell differentiation (GO:0032829)	Natural killer cell mediated cytotoxicity
Regulation of tumor necrosis factor (ligand) superfamily member 11 production (GO:2000307)	Measles
Positive regulation of gastro-intestinal system smooth muscle contraction (GO:1904306)	Malaria
Regulation of gastro-intestinal system smooth muscle contraction (GO:1904304)	Leishmaniasis
Positive regulation of eating behavior (GO:1904000)	Pertussis

Of the top 15 KEGG pathways the Neuroactive ligand-receptor interaction pathway and the cAMP signaling pathway are the most biologically relevant. Genes of interest in above pathways are: *DRD2, GHRL, GHSR, OPRM1, PTAFR, PTGER3, SSTR2*, and *TRPV1*. As indicated by the presence of the genes in these pathways and processes, the immune system can affect the motility function of the gut, which is supportive for the applied approach.

#### Predicted Effects on Extracellular Digestion of Ingested Material

The 99 genes involved in extracellular digestion of ingested material which are shared with the immune system (Figure 2, red numbers in yellow area) were used for the enrichment analysis. The top 15 GO biological processes and KEGG pathways involved in extracellular digestion/immune are listed in Table 7. The biological processes in extracellular digestion are most likely to be affected by the immune system in the cysteine biosynthetic process from serine, regulation of pancreatic juice secretion, regulation of small intestinal transit, regulation of hindgut contraction, regulation of appetite, regulation of response to food, regulation of digestive system process, cysteine biosynthetic process via cystathionine, and intestinal D-glucose absorption.

**Table 7:** Top 15 GO processes and KEGG pathways related to the selected 99 gut-immune genes involved in extracellular digestion.

**Top 15 GO processes**	**Top 15 KEGG pathways**
Cysteine biosynthetic process from serine (GO:0006535)	Vitamin digestion and absorption
Positive regulation of blood microparticle formation (GO:2000334)	Neuroactive ligand-receptor interaction
Regulation of blood microparticle formation (GO:2000332)	Rheumatoid arthritis
Positive regulation of pancreatic juice secretion (GO:0090187)	Inflammatory bowel disease (IBD)
Positive regulation of small intestinal transit (GO:0120058)	Fat digestion and absorption
Regulation of small intestinal transit (GO:0120057)	Bile secretion
Regulation of hindgut contraction (GO:0043134)	Asthma
Positive regulation of appetite (GO:0032100)	Graft-vs.-host disease
Positive regulation of response to food (GO:0032097)	cAMP signaling pathway
Regulation of gastric motility (GO:1905333)	Adipocytokine signaling pathway
Positive regulation of digestive system process (GO:0060456)	Allograft rejection
Negative regulation of gastric acid secretion (GO:0060455)	Leishmaniasis
Cysteine biosynthetic process via cystathionine (GO:0019343)	Gastric acid secretion
Ureter morphogenesis (GO:0072197)	Toxoplasmosis
Ureter smooth muscle cell differentiation (GO:0072193)	Type I diabetes mellitus

Of the top 15 KEGG pathways the vitamin digestion and absorption, fat digestion and absorption, bile secretion, adipocytokine signaling pathway, and gastric acid secretion are the most biologically relevant. Genes of interest in above pathways are: *ABCB4, ABCG5, CD36, APOA1, APOA4, AQP1, CUBN, EZR, HRH2, KCNQ1, LDLR, LEP, PPARGC1A, NPY, SCARB1, SSTR2, SST, SULT2A1, TCN2*, and *TNF*. As indicated by the presence of the genes in these pathways and processes, the immune system can affect extracellular digestion, specifically on vitamin and fat digestion and absorption as well as on the regulation of the digestive system and gastric acid in general, again providing support to the applied approach.

#### Predicted Effects on Absorption of Essential Nutrients

The 210 genes involved in the absorption of essential nutrients which are shared with the immune system (Figure 2, red numbers in green area) were used for the enrichment analysis. The top 17 GO biological processes and KEGG pathways involved in absorption essential nutrients/immune are listed in Table 8. The biological processes related to absorption of essential nutrients most likely to be affected by the immune system are in the regulation of cell proliferation involved in outflow tract morphogenesis, protein localization to bicellular tight junction, Kit signaling pathway, cysteine biosynthetic process from serine, cellular response to stem cell factor stimulus, response to stem cell factor, and intestinal D-glucose absorption.

**Table 8:** Top 17 GO processes and KEGG pathways related to the selected 210 gut-immune genes involved in absorption of essential nutrients.

**Top 17 GO processes**	**Top 15 KEGG pathways**
Regulation of cell proliferation involved in outflow tract morphogenesis (GO:1901963)	Tight junction
Kit signaling pathway (GO:0038109)	Leukocyte transendothelial migration
Cysteine biosynthetic process from serine (GO:0006535)	Cell adhesion molecules (CAMs)
Positive regulation of blood microparticle formation (GO:2000334)	Arrhythmogenic right ventricular cardiomyopathy (ARVC)
Regulation of blood microparticle formation (GO:2000332)	Adherens junction
Negative regulation of cell proliferation involved in heart morphogenesis (GO:2000137)	Endometrial cancer
Epididymis development (GO:1905867)	Rap1 signaling pathway
Detoxification of mercury ion (GO:0050787)	T cell receptor signaling pathway
Cellular response to stem cell factor stimulus (GO:0036216)	Regulation of actin cytoskeleton
Response to stem cell factor (GO:0036215)	Hepatitis C
Cellular response to methylglyoxal (GO:0097238)	Bacterial invasion of epithelial cells
Dihydrofolate metabolic process (GO:0046452)	Proteoglycans in cancer
Cellular response to indole-3-methanol (GO:0071681)	Pathogenic Escherichia coli infection
Response to indole-3-methanol (GO:0071680)	Hematopoietic cell lineage
Tolerance induction to self-antigen (GO:0002513)	Pathways in cancer
Susceptibility to natural killer cell mediated cytotoxicity (GO:0042271)	
Maintenance of gastrointestinal epithelium (GO:0030277)	

Of the top 15 KEGG pathways TJ, leukocyte transendothelial migration, cell adhesion molecules (CAMs), adherens junction and Rap1 signaling pathway are the most biologically relevant. Genes of interest in above pathways are: *ACTN4, AKT1, ASH1L, PATJ, ACTN4, CADM1, CASK, CD2, CD226, CDC42, CDH1, CDH2, CDH3, CDH5, CTNNA1, CTNNA2, CTNNA3, CTNNB1, CNTNAP2, CTNNB1, CDC42, CLDN1, CLDN11, CLDN3, CLDN4, CLDN7, CLDN9, CDC42, CDH1, CDK4, EPB41L3, EZR, FGF13, FGFR4, IGSF5, ITGA6, ITGB1, INS, IQGAP1, KIT, KRIT1, MAGI1, MAGI2, MAP2K2, NECTIN1, OCLN, PATJ, PIK3R1, PLCG1, PRKCD, PRKCZ, PTPN6, PTPRJ, PVR, RHOA, SKAP1, SYMPK, TEK, TGFBR1, TJP3, TLN1*, and *VAV1*. As indicated by the presence of the genes in these pathways and processes, the immune system can impact the barrier function of the gut as well as the absorption of nutrients.

#### Predicted Effects on Protection From Injurious or Allergenic Materials

The 680 genes involved in protection from injurious or allergenic materials which are shared with the immune system (Figure 2, red numbers in red area) were used for the enrichment analysis. The top 20 biological processes and KEGG pathways involved in protection from injurious or allergenic material/immune are listed in Table 9. The biological processes in protection most likely to be affected by the immune system are in regulation of nitrogen utilization, cellular response to triacyl bacterial lipopeptide, response to triacyl bacterial lipopeptide, toll-like receptor TLR1:TLR2 signaling pathway, fever generation, regulation of TRAIL-activated apoptotic signaling pathway, complement-mediated synapse pruning, nitric oxide transport, regulation of chronic inflammatory response to antigenic stimulus, regulation of type III interferon production, positive regulation of calcidiol 1-monooxygenase activity, positive regulation of vitamin D biosynthetic process, tolerance induction to self-antigen, negative regulation of interleukin-8 biosynthetic process, negative regulation of T-helper 2 cell cytokine production, interleukin-10 production, and activation of cysteine-type endopeptidase activity involved in apoptotic signaling pathway.

**Table 9:** Top 20 GO processes and KEGG pathways related to the selected 680 gut-immune genes involved in protection from injurious or allergenic material.

**Top 20 GO processes**	**Top 20 KEGG pathways**
Positive regulation of vitamin metabolic process (GO:0046136)	Inflammatory bowel disease (IBD)
Positive regulation of toll-like receptor 9 signaling pathway (GO:0034165)	Cytokine-cytokine receptor interaction
Tolerance induction to self-antigen (GO:0002513)	Measles
Regulation of TRAIL-activated apoptotic signaling pathway (GO:1903121)	Tuberculosis
Regulation of nitrogen utilization (GO:0006808)	Influenza A
Cellular response to triacyl bacterial lipopeptide (GO:0071727)	Herpes simplex infection
Response to triacyl bacterial lipopeptide (GO:0071725)	Hepatitis B
Negative regulation of interleukin-8 biosynthetic process (GO:0045415)	Leishmaniasis
Microglial cell activation involved in immune response (GO:0002282)	Hepatitis C
Regulation of type IIa hypersensitivity (GO:0001796)	Chagas disease (American trypanosomiasis)
Negative regulation of T-helper 2 cell cytokine production (GO:2000552)	Toxoplasmosis
Toll-like receptor TLR1:TLR2 signaling pathway (GO:0038123)	Malaria
Interleukin-10 production (GO:0032613)	Toll-like receptor signaling pathway
Epididymis development (GO:1905867)	Pathways in cancer
Negative regulation of helicase activity (GO:0051097)	Cell adhesion molecules (CAMs)
Positive regulation of TRAIL-activated apoptotic signaling pathway (GO:1903984)	Pertussis
Negative regulation of complement-dependent cytotoxicity (GO:1903660)	Chemokine signaling pathway
Regulation of complement-dependent cytotoxicity (GO:1903659)	Jak-STAT signaling pathway
Nitric oxide transport (GO:0030185)	Rheumatoid arthritis
Regulation of lung blood pressure (GO:0014916)	HIF-1 signaling pathway

Of the top 20 KEGG pathways IBD, cytokine-cytokine receptor interaction, TLR signaling pathway, pathways in cancer, CAMs, chemokine signaling pathway, Jak-STAT signaling pathway, and viral/bacterial infection related pathways [Measles, Tuberculosis, Influenza A, Herpes simplex infection, Hepatitis B, Leishmaniasis, Hepatitis C, Chagas disease (American trypanosomiasis), Toxoplasmosis, Malaria, Pertussis and Epstein-Barr virus infection] are the most biologically relevant. These pathways include many interleukins, chemokines and surface cluster molecules such as: *RELA, SMAD3, TBX21, FOXP3, IFN-GR1, IFN-G, IL-1A, IL-1B, IL-10, IL-12B, IL-13, IL-17A, IL-18, IL-2, IL-21R, IL-23R, IL-4R, IL-4, IL-5, IL-6, HLA-DPA1, HLA-DPB1, HLA-DQA1, HLA-DQB1, HLA-DRA, HLA-DRB1, STAT1, STAT3, STAT6, TLR2, TLR4, TLR5*, and *TGF-B1*. As indicated by the presence of the genes in these pathways and processes, the immune system greatly impacts the protection function of the gut.

In conclusion, with this approach 1,296 molecules were identified to be involved in gut function, of which 753 molecules are likely to be affected by nutritional immune interventions.

### Proof of Principle/*in silico* Test Case

The top interacting genes identified by the CTD for vitamin D were used for comparison with our set of 1,296 gut-function related genes, to check whether the previously described adverse/beneficial effects of vitamin D on gut function could be predicted accurately.

#### Vitamin D

**Figure 4:**
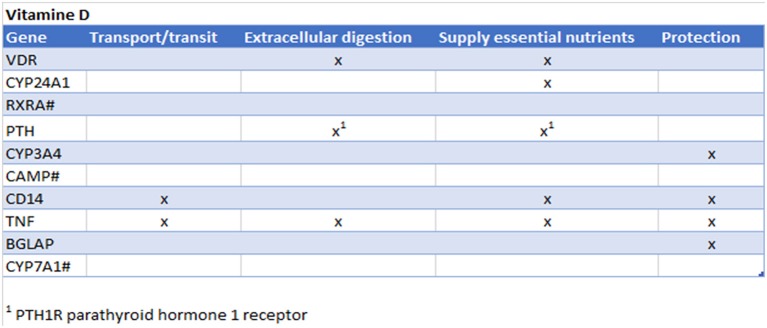
Prediction from CTD vitamin D. Gene names are listed of the top 10 interacting genes per chemical. Crosses (x) indicate in which of the four gut functions they are involved. ^#^Gene not found in all gut functions.

Vitamin D is known to play an important role in bone metabolism through regulation of calcium and phosphate homeostasis and plays an important role in immune system regulation via, for instance, the suppression of mast cell activation and IgE synthesis from B cells (1), and increase the number of tolerogenic dendritic cells and IL-10-producing regulatory T cells (2). Vitamin D is produced by the body during exposure to sunlight, but is also found in oily fish, eggs and fortified food products and is used for nutritional interventions to check their immunomodulatory properties in food allergy (3), autoimmunity (4), respiratory tract infection (5–8), and cancer (9). In addition to causing rickets, vitamin D deficiency has been linked to respiratory infections such as pneumonia, tuberculosis and bronchiolitis (5–8). Vitamin D was checked in the CTD for its top interacting genes. Subsequently, those genes were checked to see whether they were involved in the four gut functions (Figure 4) and therefore potentially influence the gut functions. Interestingly, in all four of the gut functions the top interacting genes were present.

These findings are confirmed in literature: Vitamin D plays an important role in calcium absorption, which is important for bone metabolism, but also to provide calcium to the nerve cells. In addition vitamin D has shown to protect intestinal microbiota (10–12). Adverse effects of severe hypercalcemia and hyperphoshatemia induces by exogenous vitamin D is also affecting the gastrointestinal system: including abdominal pain, anorexia, constipation, nausea, peptic ulcer, and vomiting (13, 14). Vitamin D deficiency exacerbates allergic diarrhea (15). Vitamin D is known to induce antimicrobial peptides (such as cathelicidins and defensins (16, 17) and has been shown in a clinical study that there is a direct correlation between vitamin D administration and serum cathelicidin levels in healthy subjects (18). Vitamin D is also known to enhance the gut epithelial barrier (19).

This example illustrates that the prediction with our systems biology approach is promising and can be used to find both negatively as positively correlated interactions. It must be noted that this approach is greatly influenced by the amount of data available on a specific compound; there will be more evidence on the top interacting genes when there are more data available. Therefore, it is of importance that the predicted biomarkers are properly validated in an appropriate assay/study.

## Discussion

The aim of this study was to identify crucial biomarkers for the assessment of safety and efficacy of oral immune interventions on gut function by using systems biology approaches. An enormous advantage of using these systems approaches is that all information can be collected and selected in an automated fashion, thereby enabling the possibility to screen and select large databases with genes and their biological roles/pathways. The next step would be to validate their biological role.

By making use of these comprehensive databases such as PANTHER (www.geneontology.org/) and CTD (http://ctdbase.org/) we were able to determine the overlap between gut function related genes and immune disease related genes. In total 753 genes were shared, indicating the strong relationship between the immune system and gut functions. As expected, the gut function “protection from injurious or allergenic material” (from here on briefly referred to as “protection”) shares the highest number of genes with the immune disease related genes [680 shared (63%)]. However, as with the other gut functions there is quite some overlap: transport/transit ingested material [37 shared (69%)], extracellular digestion ingested material [99 shared (40%)], and absorption of essential nutrients [210 shared (37%)].

There is a multitude of assays available to study different functions of the gut physiology. However, it appears that most assays described in literature are related to the protection, transit and/or transport. In this study, several of the identified shared genes are already measured in currently existing assays, which are mostly related to protection, transit and/or transport. It would be very interesting to include the newly identified biomarkers in the assays related to the other gut functions as they are predicted to play a role both in immune as well as a gut function, thereby indicating the putative importance of such molecule during nutritional immune intervention.

By performing a GO enrichment analysis and an enrichment analysis using DAVID bioinformatics on the gene set shared between gut function transport and transit and immune disease related genes the neuroactive ligand-receptor interaction pathway and the cAMP signaling pathway were identified. Neurotransmitters (opoid) and cAMP are known to affect smooth muscle contractions and motility and the cAMP pathway generally reduces excitability and contraction by activating K+ channels and by reducing the Ca^2+^ sensitivity of the contractile apparatus (98). Therefore, neuron expression, tyrosine kinase receptor (c-kit) and serotonin are often studied to measure motility, however it could be useful to include molecules related to G protein-coupled receptors (not found with system biology approach directly).

Current methods to assess extracellular digestion include indirect calorimetry (65), ELISA by measurement of ghrelin (66) in plasma and gas chromatography or mass spectrometry to measure SCFAs and branched fatty acids (67). It could however also be interesting to measure apolipoprotein (APO) A1 and A4 in relation to lipid transport and CD36 in fat absorption.

Methods to assess the supply of essential nutrients mostly include absorption assays and metabolic chambers (70). Parathyroid hormones might also be interesting to measure as it is the most important endocrine regulator of calcium and phosphorus concentration in extracellular fluid (99). Interestingly, a recent meta-analysis of available clinical trials indicates that 1,000 IU vitamin D supplementation can suppress serum parathyroid hormone levels, while 4,000 IU of vitamin D3 was associated with the largest increase in serum 25-hydroxyvitamin D levels in an overweight and obese population (100).

Histological examination is a common experimental way of studying aspects of barrier function, for instance immunofluorescent antibody detection of TJs (90) or adherens junctions (91), or to detect intestinal pathology including ulcerations of the mucosa and severe intestinal inflammation that will cause and contribute to increased intestinal permeability. It would be a good opportunity to combine relevant molecules with histology, so the biological validation can also be performed. For instance this assay could include molecules of one of the central signaling pathway JAK-STAT, which regulates the adaptive and innate immune arms of mucosal immunity as well as epithelial repair and regeneration (101). Epithelial repair and regeneration are important in epithelial integrity as well as gut protection function. Another opportunity for study would be to include molecules that are involved in the activation of STAT3, which stimulates antimicrobial production (102). Measurement of the molecules related to this pathway will also help to evaluate gut function. However, it must be noted that some of the mentioned assessments may only be feasible in preclinical but not in clinical settings due to their invasive nature.

CD36 was identified as an interesting molecule as it is predicted to be involved in three different gut functions: extracellular digestion, absorption of essential nutrients and protection. For example, CD36-dependent signaling mediates fatty acid-induced gut release of secretin and cholecystokinin (103), and could therefore be a good marker of extracellular digestion. Furthermore, there is a direct mechanistic link between CD36 engagement and IL-10 induction, opening up new possibilities for using CD36 ligands, agents that increase CD36 expression or a combination of both to modulate inflammation and treat, or even prevent, an important set of chronic disorders (104).

The systems biology approach described here is a promising tool, but it does have its limitations. These limitations are based on the fact that it is essential that the data used are accurate, complete and up to date. The current approach used curated data from CTD and Panther databases. As not all information available from literature has been added to these databases, it is possible that not all relationships between the immune system and gut functioning have been captured by our systems biology approach. Moreover, data are continuously generated so the approach presented here needs continuous on-going scheduled refinements and improvements. Furthermore, it might be subjected to a reporting bias as it can be difficult to distinguish the absence of a relationship between molecules/pathways from a lack of evaluation of the relationship/pathways. In addition, molecules indicated in the databases are sometimes not annotated/linked to a specific cell type/organ whereas it is known that molecules in the context of specific cell types influence certain gut functions. For instance eosinophil related proteins (28) and markers for specific T cell subsets (29) are not included in our system immunology approach, and are apparently not included in the databases. This needs some further investigation. Another limitation of the approach concerns the simplification of the complexity of the immune system and the gut functionalities. In this approach, the connectivity between the processes, genes and diseases are elucidated but the nature of these relationships (co-expression, feedback, crosstalk, activation, etc.) at each layer and between layers are disregarded. In this approach, the key markers are identified but their roles in the complexity of the biological processes are not studied.

As an appropriately functioning immune system is a dynamic system which is in balance, an upregulation/activation of certain pathways/molecules does not automatically result in a malfunction of the immune system or influence gut functions, as a healthy dynamic immune system will eventually return to a balanced state (immune resilience). Therefore, it is of utmost importance that the selected biomarkers that are anticipated to play a role in the studied gut health functions after oral immune intervention, are validated in an appropriate assay/study to understand the biological relevance of induced changes.

A next step is to prioritize and validate useful biomarkers for in nutritional intervention studies. For instance, by making use of the identified crossroads to identify which of the proteins could best be used as part of a panel of biomarkers that distinguishes whether a nutritional intervention can affect specific gut functions. This can be performed by selecting a set of common and unique markers per gut functionality. In addition, the importance of the molecule and how essential it is in specific gut functions should be checked by performing pathway analysis. Lastly, the molecules should be checked for their feasibility to be measured on both gene expression level and at protein level. This will allow the measurement of gut function upon nutritional immune intervention.

In conclusion, we describe a systems biology approach that helps to clarify on one hand the relationships between immune system and gut functions, and on the other hand the identification of candidate biomarkers to monitor effects on gut functions upon nutritional immune interventions.

## Data Availability Statement

All datasets generated for this study are included in the article/Supplementary Material.

## Author Contributions

MM, TB, RD, LV, and JB contributed conception and design of the study. TB, RD, and LV organized the database. MM and JB performed databases searches. MM, LV, and JB wrote the manuscript. All authors contributed to manuscript revision, read, and approved the submitted version.

### Conflict of Interest

JG is employed by Danone Food Safety Center. LK, LH, and KK are employed by Danone Nutricia Research. The remaining authors declare that the research was conducted in the absence of any commercial or financial relationships that could be construed as a potential conflict of interest.
