# Supplementary material for: Network-Based Selection of Candidate Markers and Assays to Assess the Impact of Oral Immune Interventions on Gut Functions
Source: Front Immunol. 2019 Nov 13;10:2672. doi: 10.3389/fimmu.2019.02672 (PMC6863931; doi:10.3389/fimmu.2019.02672)
Supplement: Supplementary file 1 [file Table_1.DOCX]

**Supplementary table I: Selected MeSH and GO terms related to gut functions.**

| **Gut functions** | **Related GO terms** | **Related MESH terms** |
| --- | --- | --- |
| 1) Transport / transit ingested material | - - GO:0120054 intestinal motility   - GO:0035482 gastric motility   - GO:0043133 hindgut contraction   - GO:0030432 peristalsis   - GO:0014831 gastro-intestinal system smooth muscle contraction   - GO:0030421 defecation | - - Constipation D003248   - Vomiting D014839   - Bile Reflux D001655   - Laryngopharyngeal Reflux D057045   - Flatulence D005414   - Abdominal Pain D015746   - Diarrhea D003967   - Nausea D009325   - Eructation D004884   - Gagging D005683   - Heartburn D006356 |
| 2) Extracellular digestion ingested material | - - GO:0007586 digestion   - GO:0050892 intestinal absorption   - GO:0006113 fermentation (NB. Related to microbe-derived products such as lactate) | - - Dyspepsia D004415   - Malabsorption Syndromes D008286   - Malnutrition D044342 |
| 3) Intracellular digestion and metabolism | - - GO:0005764 lysosome (too broad)   - GO:0044237 cellular metabolic process (too broad) | - - Dyspepsia D004415   - Malabsorption Syndromes D008286   - Malnutrition D044342 |
| 4) Excretion Into gut lumen | NO GO terms identified | NO CTD terms identified |
| 5) Supply essential nutrients | - - GO:0030277 maintenance of gastrointestinal epithelium   - GO:0005911 cell-cell junction   - GO:0050892 intestinal absorption | - - Diarrhea D003967   - Malnutrition D044342   - Water-Electrolyte Imbalance D014883 |
| 6) Protection from injurious or allergenic material | - - GO:0030277 maintenance of gastrointestinal epithelium   - GO:0005911 cell-cell junction   - GO:0070254 mucus secretion   - GO:0046541 saliva secretion | - - Hypersensitivity, Immediate D006969   - Bacterial Infections and mycosis D001423   - Virus diseases D014777   - Parasitic diseases D010272   - Gastroenteritis D005759   - Diarrhea D003967   - Vomiting D014839 |
