# Supplementary material for: Network-Based Selection of Candidate Markers and Assays to Assess the Impact of Oral Immune Interventions on Gut Functions
Source: Front Immunol. 2019 Nov 13;10:2672. doi: 10.3389/fimmu.2019.02672 (PMC6863931; doi:10.3389/fimmu.2019.02672)
Supplement: Supplementary file 2 [file Table_2.DOCX]

**Supplementary table II: Genes involved in transport/transit**

| **EntrezID** | **Name** |
| --- | --- |
| 929 | CD14 |
| 1440 | CSF3 |
| 1813 | DRD2 |
| 1890 | TYMP |
| 2316 | FLNA |
| 2641 | GCG |
| 2668 | GDNF |
| 2693 | GHSR |
| 2796 | GNRH1 |
| 2984 | GUCY2C |
| 3440 | IFNA2 |
| 3458 | IFNG |
| 3552 | IL1A |
| 3557 | IL1RN |
| 3815 | KIT |
| 4057 | LTF |
| 4072 | EPCAM |
| 4192 | MDK |
| 4645 | MYO5B |
| 4988 | OPRM1 |
| 5020 | OXT |
| 5243 | ABCB1 |
| 5443 | POMC |
| 5444 | PON1 |
| 5724 | PTAFR |
| 5733 | PTGER3 |
| 6752 | SSTR2 |
| 7124 | TNF |
| 7442 | TRPV1 |
| 8573 | CASK |
| 8797 | TNFRSF10A |
| 9968 | MED12 |
| 10653 | SPINT2 |
| 23213 | SULF1 |
| 50943 | FOXP3 |
| 51738 | GHRL |
| 374569 | ASPG |
