# Supplementary material for: Network-Based Selection of Candidate Markers and Assays to Assess the Impact of Oral Immune Interventions on Gut Functions
Source: Front Immunol. 2019 Nov 13;10:2672. doi: 10.3389/fimmu.2019.02672 (PMC6863931; doi:10.3389/fimmu.2019.02672)
Supplement: Supplementary file 3 [file Table_3.DOCX]

**Supplementary table III: Genes involved in extracellular digestion**

| **EntrezID** | **Name** |
| --- | --- |
| 12 | SERPINA3 |
| 48 | ACO1 |
| 142 | PARP1 |
| 335 | APOA1 |
| 337 | APOA4 |
| 358 | AQP1 |
| 652 | BMP4 |
| 847 | CAT |
| 864 | RUNX3 |
| 875 | CBS |
| 886 | CCKAR |
| 948 | CD36 |
| 949 | SCARB1 |
| 1184 | CLCN5 |
| 1233 | CCR4 |
| 1314 | COPA |
| 1356 | CP |
| 1493 | CTLA4 |
| 1636 | ACE |
| 1645 | AKR1C1 |
| 1646 | AKR1C2 |
| 1674 | DES |
| 1719 | DHFR |
| 1890 | TYMP |
| 2113 | ETS1 |
| 2255 | FGF10 |
| 2524 | FUT2 |
| 2693 | GHSR |
| 2984 | GUCY2C |
| 3115 | HLA-DPB1 |
| 3117 | HLA-DQA2 |
| 3119 | HLA-DQB1 |
| 3274 | HRH2 |
| 3600 | IL15 |
| 3784 | KCNQ1 |
| 3949 | LDLR |
| 3952 | LEP |
| 4036 | LRP2 |
| 4072 | EPCAM |
| 4128 | MAOA |
| 4224 | MEP1A |
| 4524 | MTHFR |
| 4548 | MTR |
| 4552 | MTRR |
| 4583 | N/A |
| 4585 | MUC4 |
| 4588 | MUC6 |
| 4645 | MYO5B |
| 4852 | NPY |
| 4883 | NPR3 |
| 5020 | OXT |
| 5021 | OXTR |
| 5027 | P2RX7 |
| 5244 | ABCB4 |
| 5265 | SERPINA1 |
| 5357 | PLS1 |
| 5444 | PON1 |
| 5646 | PRSS3 |
| 5733 | PTGER3 |
| 5745 | PTH1R |
| 5826 | ABCD4 |
| 5950 | RBP4 |
| 6343 | SCT |
| 6446 | SGK1 |
| 6514 | SLC2A2 |
| 6518 | SLC2A5 |
| 6584 | SLC22A5 |
| 6647 | SOD1 |
| 6662 | SOX9 |
| 6750 | SST |
| 6751 | SSTR1 |
| 6752 | SSTR2 |
| 6822 | SULT2A1 |
| 6948 | TCN2 |
| 7031 | TFF1 |
| 7052 | TGM2 |
| 7086 | TKT |
| 7099 | TLR4 |
| 7124 | TNF |
| 7376 | NR1H2 |
| 7421 | VDR |
| 7430 | EZR |
| 7442 | TRPV1 |
| 8029 | CUBN |
| 8764 | TNFRSF14 |
| 10891 | PPARGC1A |
| 11171 | STRAP |
| 27232 | GNMT |
| 29108 | PYCARD |
| 51738 | GHRL |
| 54106 | TLR9 |
| 54658 | UGT1A1 |
| 56287 | GKN1 |
| 57016 | AKR1B10 |
| 57178 | ZMIZ1 |
| 59067 | IL21 |
| 60468 | BACH2 |
| 64081 | PBLD |
| 64240 | ABCG5 |
