# Supplementary material for: Network-Based Selection of Candidate Markers and Assays to Assess the Impact of Oral Immune Interventions on Gut Functions
Source: Front Immunol. 2019 Nov 13;10:2672. doi: 10.3389/fimmu.2019.02672 (PMC6863931; doi:10.3389/fimmu.2019.02672)
Supplement: Supplementary file 4 [file Table_4.DOCX]

**Supplementary table IV: genes involved in absorption essential nutrients**

| **EntrezID** | **Name** |
| --- | --- |
| 12 | SERPINA3 |
| 48 | ACO1 |
| 81 | ACTN4 |
| 117 | ADCYAP1R1 |
| 118 | ADD1 |
| 142 | PARP1 |
| 154 | ADRB2 |
| 183 | AGT |
| 207 | AKT1 |
| 231 | AKR1B1 |
| 301 | ANXA1 |
| 302 | ANXA2 |
| 324 | APC |
| 335 | APOA1 |
| 337 | APOA4 |
| 360 | AQP3 |
| 387 | RHOA |
| 476 | ATP1A1 |
| 481 | ATP1B1 |
| 551 | AVP |
| 595 | CCND1 |
| 634 | CEACAM1 |
| 652 | BMP4 |
| 659 | BMPR2 |
| 794 | CALB2 |
| 796 | CALCA |
| 847 | CAT |
| 875 | CBS |
| 889 | KRIT1 |
| 914 | CD2 |
| 916 | CD3E |
| 929 | CD14 |
| 948 | CD36 |
| 949 | SCARB1 |
| 998 | CDC42 |
| 999 | CDH1 |
| 1000 | CDH2 |
| 1001 | CDH3 |
| 1003 | CDH5 |
| 1019 | CDK4 |
| 1072 | CFL1 |
| 1184 | CLCN5 |
| 1244 | ABCC2 |
| 1265 | CNN2 |
| 1266 | CNN3 |
| 1356 | CP |
| 1364 | CLDN4 |
| 1365 | CLDN3 |
| 1366 | CLDN7 |
| 1440 | CSF3 |
| 1445 | CSK |
| 1495 | CTNNA1 |
| 1496 | CTNNA2 |
| 1499 | CTNNB1 |
| 1586 | CYP17A1 |
| 1591 | CYP24A1 |
| 1605 | DAG1 |
| 1645 | AKR1C1 |
| 1674 | DES |
| 1719 | DHFR |
| 1823 | DSC1 |
| 1825 | DSC3 |
| 2173 | FABP7 |
| 2195 | FAT1 |
| 2196 | FAT2 |
| 2258 | FGF13 |
| 2264 | FGFR4 |
| 2316 | FLNA |
| 2524 | FUT2 |
| 2697 | GJA1 |
| 2701 | GJA4 |
| 2705 | GJB1 |
| 2706 | GJB2 |
| 2709 | GJB5 |
| 2908 | NR3C1 |
| 2984 | GUCY2C |
| 2995 | GYPC |
| 3249 | HPN |
| 3440 | IFNA2 |
| 3458 | IFNG |
| 3552 | IL1A |
| 3557 | IL1RN |
| 3569 | IL6 |
| 3611 | ILK |
| 3630 | INS |
| 3655 | ITGA6 |
| 3678 | ITGA5 |
| 3688 | ITGB1 |
| 3728 | JUP |
| 3767 | KCNJ11 |
| 3784 | KCNQ1 |
| 3815 | KIT |
| 3856 | KRT8 |
| 3875 | KRT18 |
| 3949 | LDLR |
| 3952 | LEP |
| 4036 | LRP2 |
| 4057 | LTF |
| 4067 | LYN |
| 4072 | EPCAM |
| 4092 | SMAD7 |
| 4128 | MAOA |
| 4524 | MTHFR |
| 4548 | MTR |
| 4552 | MTRR |
| 4583 | N/A |
| 4585 | MUC4 |
| 4588 | MUC6 |
| 4645 | MYO5B |
| 4982 | TNFRSF11B |
| 5010 | CLDN11 |
| 5020 | OXT |
| 5021 | OXTR |
| 5027 | P2RX7 |
| 5058 | PAK1 |
| 5239 | PGM5 |
| 5244 | ABCB4 |
| 5265 | SERPINA1 |
| 5295 | PIK3R1 |
| 5317 | PKP1 |
| 5335 | PLCG1 |
| 5357 | PLS1 |
| 5420 | PODXL |
| 5443 | POMC |
| 5444 | PON1 |
| 5580 | PRKCD |
| 5590 | PRKCZ |
| 5605 | MAP2K2 |
| 5744 | PTHLH |
| 5745 | PTH1R |
| 5756 | TWF1 |
| 5777 | PTPN6 |
| 5795 | PTPRJ |
| 5796 | PTPRK |
| 5817 | PVR |
| 5818 | NECTIN1 |
| 5826 | ABCD4 |
| 5950 | RBP4 |
| 6513 | SLC2A1 |
| 6514 | SLC2A2 |
| 6518 | SLC2A5 |
| 6521 | SLC4A1 |
| 6584 | SLC22A5 |
| 6647 | SOD1 |
| 6662 | SOX9 |
| 6750 | SST |
| 6905 | TBCE |
| 6948 | TCN2 |
| 7010 | TEK |
| 7031 | TFF1 |
| 7046 | TGFBR1 |
| 7086 | TKT |
| 7094 | TLN1 |
| 7099 | TLR4 |
| 7124 | TNF |
| 7317 | UBA1 |
| 7409 | VAV1 |
| 7421 | VDR |
| 7430 | EZR |
| 8029 | CUBN |
| 8189 | SYMPK |
| 8573 | CASK |
| 8600 | TNFSF11 |
| 8631 | SKAP1 |
| 8647 | ABCB11 |
| 8825 | LIN7A |
| 8826 | IQGAP1 |
| 9076 | CLDN1 |
| 9080 | CLDN9 |
| 9223 | MAGI1 |
| 9863 | MAGI2 |
| 10058 | ABCB6 |
| 10207 | PATJ |
| 10211 | FLOT1 |
| 10397 | NDRG1 |
| 10653 | SPINT2 |
| 10666 | CD226 |
| 10809 | STARD10 |
| 11151 | CORO1A |
| 11171 | STRAP |
| 11187 | PKP3 |
| 11252 | PACSIN2 |
| 11315 | PARK7 |
| 23136 | EPB41L3 |
| 23209 | MLC1 |
| 23586 | DDX58 |
| 23705 | CADM1 |
| 25932 | CLIC4 |
| 26047 | CNTNAP2 |
| 27134 | TJP3 |
| 27232 | GNMT |
| 29108 | PYCARD |
| 29119 | CTNNA3 |
| 30011 | SH3KBP1 |
| 50943 | FOXP3 |
| 51599 | LSR |
| 54106 | TLR9 |
| 55870 | ASH1L |
| 55971 | BAIAP2L1 |
| 57449 | PLEKHG5 |
| 57731 | SPTBN4 |
| 64081 | PBLD |
| 64240 | ABCG5 |
| 79026 | AHNAK |
| 84962 | AJUBA |
| 92140 | MTDH |
| 150084 | IGSF5 |
| 201163 | FLCN |
| 259197 | NCR3 |
| 100506658 | OCLN |
