# Supplementary material for: Network-Based Selection of Candidate Markers and Assays to Assess the Impact of Oral Immune Interventions on Gut Functions
Source: Front Immunol. 2019 Nov 13;10:2672. doi: 10.3389/fimmu.2019.02672 (PMC6863931; doi:10.3389/fimmu.2019.02672)
Supplement: Supplementary file 5 [file Table_5.DOCX]

**Supplementary table V: genes involved in protection from injurious or allergenic material**

| **EntrezID** | **Name** |
| --- | --- |
| 12 | SERPINA3 |
| 81 | ACTN4 |
| 100 | ADA |
| 108 | ADCY2 |
| 113 | ADCY7 |
| 117 | ADCYAP1R1 |
| 118 | ADD1 |
| 142 | PARP1 |
| 154 | ADRB2 |
| 196 | AHR |
| 207 | AKT1 |
| 213 | ALB |
| 217 | ALDH2 |
| 231 | AKR1B1 |
| 240 | ALOX5 |
| 301 | ANXA1 |
| 302 | ANXA2 |
| 324 | APC |
| 326 | AIRE |
| 329 | BIRC2 |
| 330 | BIRC3 |
| 335 | APOA1 |
| 355 | FAS |
| 358 | AQP1 |
| 360 | AQP3 |
| 374 | AREG |
| 383 | ARG1 |
| 384 | ARG2 |
| 387 | RHOA |
| 393 | ARHGAP4 |
| 396 | ARHGDIA |
| 397 | ARHGDIB |
| 467 | ATF3 |
| 476 | ATP1A1 |
| 481 | ATP1B1 |
| 581 | BAX |
| 595 | CCND1 |
| 596 | BCL2 |
| 632 | BGLAP |
| 634 | CEACAM1 |
| 639 | PRDM1 |
| 659 | BMPR2 |
| 710 | SERPING1 |
| 715 | C1R |
| 716 | C1S |
| 718 | C3 |
| 731 | C8A |
| 732 | C8B |
| 735 | C9 |
| 794 | CALB2 |
| 836 | CASP3 |
| 841 | CASP8 |
| 847 | CAT |
| 889 | KRIT1 |
| 914 | CD2 |
| 916 | CD3E |
| 920 | CD4 |
| 929 | CD14 |
| 940 | CD28 |
| 948 | CD36 |
| 949 | SCARB1 |
| 975 | CD81 |
| 998 | CDC42 |
| 999 | CDH1 |
| 1000 | CDH2 |
| 1001 | CDH3 |
| 1003 | CDH5 |
| 1015 | CDH17 |
| 1019 | CDK4 |
| 1026 | CDKN1A |
| 1027 | CDKN1B |
| 1072 | CFL1 |
| 1116 | CHI3L1 |
| 1154 | CISH |
| 1192 | CLIC1 |
| 1232 | CCR3 |
| 1234 | CCR5 |
| 1235 | CCR6 |
| 1241 | LTB4R |
| 1244 | ABCC2 |
| 1265 | CNN2 |
| 1266 | CNN3 |
| 1356 | CP |
| 1364 | CLDN4 |
| 1365 | CLDN3 |
| 1366 | CLDN7 |
| 1373 | CPS1 |
| 1378 | CR1 |
| 1401 | CRP |
| 1437 | CSF2 |
| 1440 | CSF3 |
| 1445 | CSK |
| 1469 | CST1 |
| 1490 | CTGF |
| 1495 | CTNNA1 |
| 1496 | CTNNA2 |
| 1499 | CTNNB1 |
| 1524 | CX3CR1 |
| 1540 | CYLD |
| 1548 | CYP2A6 |
| 1576 | CYP3A4 |
| 1605 | DAG1 |
| 1636 | ACE |
| 1647 | GADD45A |
| 1670 | DEFA5 |
| 1673 | DEFB4A |
| 1674 | DES |
| 1675 | CFD |
| 1718 | DHCR24 |
| 1728 | NQO1 |
| 1767 | DNAH5 |
| 1786 | DNMT1 |
| 1788 | DNMT3A |
| 1823 | DSC1 |
| 1825 | DSC3 |
| 1839 | HBEGF |
| 1888 | N/A |
| 1906 | EDN1 |
| 1973 | EIF4A1 |
| 1978 | EIF4EBP1 |
| 2023 | ENO1 |
| 2147 | F2 |
| 2150 | F2RL1 |
| 2161 | F12 |
| 2173 | FABP7 |
| 2195 | FAT1 |
| 2196 | FAT2 |
| 2205 | FCER1A |
| 2206 | MS4A2 |
| 2208 | FCER2 |
| 2212 | FCGR2A |
| 2213 | FCGR2B |
| 2255 | FGF10 |
| 2258 | FGF13 |
| 2264 | FGFR4 |
| 2312 | FLG |
| 2316 | FLNA |
| 2335 | FN1 |
| 2524 | FUT2 |
| 2532 | ACKR1 |
| 2537 | IFI6 |
| 2633 | GBP1 |
| 2638 | GC |
| 2641 | GCG |
| 2697 | GJA1 |
| 2701 | GJA4 |
| 2705 | GJB1 |
| 2706 | GJB2 |
| 2709 | GJB5 |
| 2720 | GLB1 |
| 2778 | GNAS |
| 2796 | GNRH1 |
| 2833 | CXCR3 |
| 2919 | CXCL1 |
| 2932 | GSK3B |
| 2934 | GSN |
| 2944 | GSTM1 |
| 2950 | GSTP1 |
| 2971 | GTF3A |
| 2984 | GUCY2C |
| 2993 | GYPA |
| 2995 | GYPC |
| 3043 | HBB |
| 3068 | HDGF |
| 3075 | CFH |
| 3077 | HFE |
| 3082 | HGF |
| 3106 | HLA-B |
| 3107 | HLA-C |
| 3113 | HLA-DPA1 |
| 3115 | HLA-DPB1 |
| 3117 | HLA-DQA1 |
| 3119 | HLA-DQB1 |
| 3122 | HLA-DRA |
| 3123 | HLA-DRB1 |
| 3135 | HLA-G |
| 3146 | HMGB1 |
| 3156 | HMGCR |
| 3162 | HMOX1 |
| 3176 | HNMT |
| 3240 | HP |
| 3249 | HPN |
| 3269 | HRH1 |
| 3291 | HSD11B2 |
| 3309 | HSPA5 |
| 3313 | HSPA9 |
| 3315 | HSPB1 |
| 3320 | HSP90AA1 |
| 3326 | HSP90AB1 |
| 3329 | HSPD1 |
| 3371 | TNC |
| 3383 | ICAM1 |
| 3399 | ID3 |
| 3429 | IFI27 |
| 3430 | IFI35 |
| 3431 | SP110 |
| 3433 | IFIT2 |
| 3434 | IFIT1 |
| 3437 | IFIT3 |
| 3439 | IFNA1 |
| 3440 | IFNA2 |
| 3454 | IFNAR1 |
| 3455 | IFNAR2 |
| 3456 | IFNB1 |
| 3458 | IFNG |
| 3459 | IFNGR1 |
| 3479 | IGF1 |
| 3552 | IL1A |
| 3553 | IL1B |
| 3557 | IL1RN |
| 3558 | IL2 |
| 3559 | IL2RA |
| 3565 | IL4 |
| 3566 | IL4R |
| 3567 | IL5 |
| 3569 | IL6 |
| 3575 | IL7R |
| 3576 | CXCL8 |
| 3577 | CXCR1 |
| 3579 | CXCR2 |
| 3586 | IL10 |
| 3587 | IL10RA |
| 3588 | IL10RB |
| 3593 | IL12B |
| 3596 | IL13 |
| 3600 | IL15 |
| 3605 | IL17A |
| 3606 | IL18 |
| 3608 | ILF2 |
| 3611 | ILK |
| 3627 | CXCL10 |
| 3655 | ITGA6 |
| 3663 | IRF5 |
| 3665 | IRF7 |
| 3676 | ITGA4 |
| 3678 | ITGA5 |
| 3683 | ITGAL |
| 3684 | ITGAM |
| 3688 | ITGB1 |
| 3692 | EIF6 |
| 3694 | ITGB6 |
| 3717 | JAK2 |
| 3728 | JUP |
| 3767 | KCNJ11 |
| 3811 | KIR3DL1 |
| 3815 | KIT |
| 3827 | KNG1 |
| 3837 | KPNB1 |
| 3845 | KRAS |
| 3856 | KRT8 |
| 3875 | KRT18 |
| 3880 | KRT19 |
| 3949 | LDLR |
| 3952 | LEP |
| 3953 | LEPR |
| 4000 | LMNA |
| 4015 | LOX |
| 4017 | LOXL2 |
| 4023 | LPL |
| 4046 | LSP1 |
| 4047 | LSS |
| 4049 | LTA |
| 4055 | LTBR |
| 4056 | LTC4S |
| 4057 | LTF |
| 4061 | LY6E |
| 4067 | LYN |
| 4072 | EPCAM |
| 4088 | SMAD3 |
| 4089 | SMAD4 |
| 4092 | SMAD7 |
| 4150 | MAZ |
| 4153 | MBL2 |
| 4159 | MC3R |
| 4170 | MCL1 |
| 4179 | CD46 |
| 4193 | MDM2 |
| 4277 | MICB |
| 4282 | MIF |
| 4313 | MMP2 |
| 4318 | MMP9 |
| 4319 | MMP10 |
| 4353 | MPO |
| 4524 | MTHFR |
| 4583 | N/A |
| 4585 | MUC4 |
| 4588 | MUC6 |
| 4589 | MUC7 |
| 4599 | MX1 |
| 4600 | MX2 |
| 4602 | MYB |
| 4609 | MYC |
| 4645 | MYO5B |
| 4803 | NGF |
| 4843 | NOS2 |
| 4846 | NOS3 |
| 4852 | NPY |
| 4864 | NPC1 |
| 4927 | NUP88 |
| 4938 | OAS1 |
| 4939 | OAS2 |
| 4940 | OAS3 |
| 4953 | ODC1 |
| 4988 | OPRM1 |
| 5010 | CLDN11 |
| 5017 | OVOL1 |
| 5021 | OXTR |
| 5027 | P2RX7 |
| 5052 | PRDX1 |
| 5054 | SERPINE1 |
| 5058 | PAK1 |
| 5071 | PRKN |
| 5087 | PBX1 |
| 5091 | PC |
| 5142 | PDE4B |
| 5144 | PDE4D |
| 5176 | SERPINF1 |
| 5178 | PEG3 |
| 5199 | CFP |
| 5239 | PGM5 |
| 5243 | ABCB1 |
| 5244 | ABCB4 |
| 5265 | SERPINA1 |
| 5295 | PIK3R1 |
| 5317 | PKP1 |
| 5321 | PLA2G4A |
| 5327 | PLAT |
| 5328 | PLAU |
| 5335 | PLCG1 |
| 5338 | PLD2 |
| 5359 | PLSCR1 |
| 5420 | PODXL |
| 5443 | POMC |
| 5465 | PPARA |
| 5468 | PPARG |
| 5479 | PPIB |
| 5515 | PPP2CA |
| 5578 | PRKCA |
| 5580 | PRKCD |
| 5581 | PRKCE |
| 5587 | PRKD1 |
| 5590 | PRKCZ |
| 5594 | MAPK1 |
| 5595 | MAPK3 |
| 5599 | MAPK8 |
| 5605 | MAP2K2 |
| 5610 | EIF2AK2 |
| 5663 | PSEN1 |
| 5685 | PSMA4 |
| 5686 | PSMA5 |
| 5687 | PSMA6 |
| 5692 | PSMB4 |
| 5699 | PSMB10 |
| 5702 | PSMC3 |
| 5705 | PSMC5 |
| 5709 | PSMD3 |
| 5714 | PSMD8 |
| 5719 | PSMD13 |
| 5720 | PSME1 |
| 5721 | PSME2 |
| 5727 | PTCH1 |
| 5728 | PTEN |
| 5729 | PTGDR |
| 5732 | PTGER2 |
| 5739 | PTGIR |
| 5742 | PTGS1 |
| 5743 | PTGS2 |
| 5756 | TWF1 |
| 5771 | PTPN2 |
| 5777 | PTPN6 |
| 5788 | PTPRC |
| 5795 | PTPRJ |
| 5796 | PTPRK |
| 5803 | PTPRZ1 |
| 5817 | PVR |
| 5818 | NECTIN1 |
| 5896 | RAG1 |
| 5901 | RAN |
| 5902 | RANBP1 |
| 5903 | RANBP2 |
| 5950 | RBP4 |
| 5970 | RELA |
| 5979 | RET |
| 6037 | RNASE3 |
| 6092 | ROBO2 |
| 6279 | S100A8 |
| 6334 | SCN8A |
| 6335 | SCN9A |
| 6347 | CCL2 |
| 6348 | CCL3 |
| 6349 | CCL3L3 |
| 6351 | CCL4 |
| 6352 | CCL5 |
| 6355 | CCL8 |
| 6356 | CCL11 |
| 6361 | CCL17 |
| 6364 | CCL20 |
| 6367 | CCL22 |
| 6369 | CCL24 |
| 6387 | CXCL12 |
| 6401 | SELE |
| 6403 | SELP |
| 6421 | SFPQ |
| 6422 | SFRP1 |
| 6423 | SFRP2 |
| 6426 | SRSF1 |
| 6484 | ST3GAL4 |
| 6513 | SLC2A1 |
| 6514 | SLC2A2 |
| 6521 | SLC4A1 |
| 6554 | SLC10A1 |
| 6556 | SLC11A1 |
| 6581 | SLC22A3 |
| 6583 | SLC22A4 |
| 6584 | SLC22A5 |
| 6597 | SMARCA4 |
| 6614 | SIGLEC1 |
| 6634 | SNRPD3 |
| 6647 | SOD1 |
| 6648 | SOD2 |
| 6662 | SOX9 |
| 6701 | SPRR2B |
| 6714 | SRC |
| 6722 | SRF |
| 6772 | STAT1 |
| 6774 | STAT3 |
| 6778 | STAT6 |
| 6845 | VAMP7 |
| 6880 | TAF9 |
| 6905 | TBCE |
| 6915 | TBXA2R |
| 6929 | TCF3 |
| 6932 | TCF7 |
| 7010 | TEK |
| 7018 | TF |
| 7031 | TFF1 |
| 7037 | TFRC |
| 7039 | TGFA |
| 7040 | TGFB1 |
| 7046 | TGFBR1 |
| 7078 | TIMP3 |
| 7094 | TLN1 |
| 7096 | TLR1 |
| 7097 | TLR2 |
| 7098 | TLR3 |
| 7099 | TLR4 |
| 7100 | TLR5 |
| 7124 | TNF |
| 7130 | TNFAIP6 |
| 7157 | TP53 |
| 7203 | CCT3 |
| 7263 | TST |
| 7266 | DNAJC7 |
| 7297 | TYK2 |
| 7305 | TYROBP |
| 7307 | U2AF1 |
| 7317 | UBA1 |
| 7332 | UBE2L3 |
| 7378 | UPP1 |
| 7409 | VAV1 |
| 7412 | VCAM1 |
| 7422 | VEGFA |
| 7442 | TRPV1 |
| 7503 | XIST |
| 7512 | XPNPEP2 |
| 7528 | YY1 |
| 7782 | SLC30A4 |
| 7852 | CXCR4 |
| 7941 | PLA2G7 |
| 8174 | MADCAM1 |
| 8189 | SYMPK |
| 8289 | ARID1A |
| 8517 | IKBKG |
| 8573 | CASK |
| 8631 | SKAP1 |
| 8638 | OASL |
| 8639 | AOC3 |
| 8647 | ABCB11 |
| 8662 | EIF3B |
| 8673 | VAMP8 |
| 8683 | SRSF9 |
| 8743 | TNFSF10 |
| 8784 | TNFRSF18 |
| 8825 | LIN7A |
| 8826 | IQGAP1 |
| 8875 | VNN2 |
| 8876 | VNN1 |
| 8877 | SPHK1 |
| 8900 | CCNA1 |
| 8924 | HERC2 |
| 8929 | PHOX2B |
| 8989 | TRPA1 |
| 9076 | CLDN1 |
| 9080 | CLDN9 |
| 9173 | IL1RL1 |
| 9223 | MAGI1 |
| 9230 | RAB11B |
| 9246 | UBE2L6 |
| 9370 | ADIPOQ |
| 9376 | SLC22A8 |
| 9531 | BAG3 |
| 9547 | CXCL14 |
| 9636 | ISG15 |
| 9861 | PSMD6 |
| 9863 | MAGI2 |
| 9971 | NR1H4 |
| 9997 | SCO2 |
| 10111 | RAD50 |
| 10135 | NAMPT |
| 10189 | ALYREF |
| 10197 | PSME3 |
| 10204 | NUTF2 |
| 10207 | PATJ |
| 10211 | FLOT1 |
| 10269 | ZMPSTE24 |
| 10280 | SIGMAR1 |
| 10297 | APC2 |
| 10318 | TNIP1 |
| 10320 | IKZF1 |
| 10346 | TRIM22 |
| 10397 | NDRG1 |
| 10410 | IFITM3 |
| 10411 | RAPGEF3 |
| 10436 | EMG1 |
| 10516 | FBLN5 |
| 10527 | IPO7 |
| 10551 | AGR2 |
| 10561 | IFI44 |
| 10572 | SIVA1 |
| 10574 | CCT7 |
| 10594 | PRPF8 |
| 10631 | POSTN |
| 10653 | SPINT2 |
| 10666 | CD226 |
| 10672 | GNA13 |
| 10693 | CCT6B |
| 10747 | MASP2 |
| 10800 | CYSLTR1 |
| 10808 | HSPH1 |
| 10809 | STARD10 |
| 10841 | FTCD |
| 10878 | CFHR3 |
| 10919 | EHMT2 |
| 10946 | SF3A3 |
| 10964 | IFI44L |
| 10980 | COPS6 |
| 10981 | RAB32 |
| 11005 | SPINK5 |
| 11127 | KIF3A |
| 11151 | CORO1A |
| 11171 | STRAP |
| 11186 | RASSF1 |
| 11187 | PKP3 |
| 11213 | IRAK3 |
| 11251 | PTGDR2 |
| 11252 | PACSIN2 |
| 11277 | TREX1 |
| 11315 | PARK7 |
| 11322 | TMC6 |
| 16190 | N/A |
| 17748 | N/A |
| 17750 | N/A |
| 18669 | N/A |
| 20305 | N/A |
| 20308 | N/A |
| 22806 | IKZF3 |
| 22891 | ZNF365 |
| 22948 | CCT5 |
| 23098 | SARM1 |
| 23136 | EPB41L3 |
| 23198 | PSME4 |
| 23209 | MLC1 |
| 23385 | NCSTN |
| 23411 | SIRT1 |
| 23424 | TDRD7 |
| 23516 | SLC39A14 |
| 23586 | DDX58 |
| 23601 | CLEC5A |
| 23705 | CADM1 |
| 24138 | IFIT5 |
| 24300 | N/A |
| 25932 | CLIC4 |
| 26047 | CNTNAP2 |
| 26191 | PTPN22 |
| 26762 | HAVCR1 |
| 27074 | LAMP3 |
| 27113 | BBC3 |
| 27134 | TJP3 |
| 27164 | SALL3 |
| 27173 | SLC39A1 |
| 27348 | TOR1B |
| 29119 | CTNNA3 |
| 30009 | TBX21 |
| 30011 | SH3KBP1 |
| 30835 | CD209 |
| 50615 | IL21R |
| 50807 | ASAP1 |
| 50943 | FOXP3 |
| 51084 | CRYL1 |
| 51131 | PHF11 |
| 51135 | IRAK4 |
| 51164 | DCTN4 |
| 51191 | HERC5 |
| 51196 | PLCE1 |
| 51599 | LSR |
| 51738 | GHRL |
| 53947 | A4GALT |
| 54106 | TLR9 |
| 54209 | TREM2 |
| 54739 | XAF1 |
| 54809 | SAMD9 |
| 54877 | ZCCHC2 |
| 54894 | RNF43 |
| 55008 | HERC6 |
| 55509 | BATF3 |
| 55601 | DDX60 |
| 55630 | SLC39A4 |
| 55714 | TENM3 |
| 55851 | PSENEN |
| 55870 | ASH1L |
| 55876 | GSDMB |
| 55971 | BAIAP2L1 |
| 56946 | EMSY |
| 57105 | CYSLTR2 |
| 57449 | PLEKHG5 |
| 57731 | SPTBN4 |
| 58508 | KMT2C |
| 60468 | BACH2 |
| 64081 | PBLD |
| 64108 | RTP4 |
| 64135 | IFIH1 |
| 64170 | CARD9 |
| 64411 | ARAP3 |
| 64761 | PARP12 |
| 79026 | AHNAK |
| 80273 | GRPEL1 |
| 80830 | APOL6 |
| 81622 | UNC93B1 |
| 84433 | CARD11 |
| 84962 | AJUBA |
| 85363 | TRIM5 |
| 85480 | TSLP |
| 90865 | IL33 |
| 91543 | RSAD2 |
| 92140 | MTDH |
| 94103 | ORMDL3 |
| 111654 | N/A |
| 114548 | NLRP3 |
| 114609 | TIRAP |
| 117156 | SCGB3A2 |
| 117289 | TAGAP |
| 134430 | WDR36 |
| 135656 | DPCR1 |
| 147138 | TMC8 |
| 148022 | TICAM1 |
| 149233 | IL23R |
| 149628 | PYHIN1 |
| 150084 | IGSF5 |
| 151887 | CCDC80 |
| 201163 | FLCN |
| 201626 | PDE12 |
| 222256 | CDHR3 |
| 259197 | NCR3 |
| 282617 | IFNL3 |
| 283234 | CCDC88B |
| 284382 | ACTL9 |
| 345611 | IRGM |
| 374569 | ASPG |
| 386653 | IL31 |
| 387129 | NPSR1 |
| 406885 | MIRLET7C |
| 406892 | MIR100 |
| 406906 | MIR122 |
| 406965 | MIR190A |
| 407055 | MIR99A |
| 440854 | CAPN14 |
| 678669 | N/A |
| 100156830 | N/A |
| 100506658 | OCLN |
| 101801591 | N/A |
